# Effects of Generative AI–Supported Virtual Reality Dental Skill Training on Learning Performance, Visual Behavior, and Cortical Activation Among Dental Students: Stratified Randomized Controlled Trial

**DOI:** 10.2196/90938

**Published:** 2026-10-01

**Authors:** Yuting Wen, Chang Wen, Siyu Huang, Yufei Chong, Dong Yang

**Affiliations:** 1 State Key Laboratory of Oral & Maxillofacial Reconstruction and Regeneration, Key Laboratory of Oral Biomedicine Ministry of Education, Hubei Key Laboratory of Stomatology School & Hospital of Stomatology Wuhan University Wuhan, Hubei China; 2 Department of Periodontology School & Hospital of Stomatology Wuhan University Wuhan, Hubei China; 3 Center for Orthodontics and Pediatric Dentistry at Optics Valley Branch School & Hospital of Stomatology Wuhan University Wuhan, Hubei China; 4 The Affilated Hospital of Hubei Provincial Government Wuhan, Hubei China

**Keywords:** generative AI, medical education, clinical skills, visual-spatial ability, cognitive load, self-regulated learning

## Abstract

**Background:**

The rapid advancement of AI, particularly generative AI (GenAI), is reshaping educational paradigms. However, its utility in preclinical digital medical skills education remains insufficiently explored.

**Objective:**

This study aims to investigate whether GenAI‑assisted training yields superior performance relative to traditional instruction in virtual reality (VR)–supported preclinical dental skill education.

**Methods:**

A total of 123 eligible students from a top‑tier Chinese university completed the trial. Participants were stratified by baseline theoretical scores and randomly assigned within each stratum into two arms: the GenAI-assisted group (n=62) received ChatGPT guidance without human tutoring, with research assistants monitoring AI outputs solely for safety and error checking; the teacher-led control group (n=61) received on-site instructor guidance. Both groups underwent standardized VR-based dental skill training for 7 days. Assessments included dental operative skill examinations, functional near-infrared spectroscopy recording, continuous eye-tracking monitoring, visual-spatial ability tests, and questionnaires measuring self-regulated learning, learning emotions, and engagement.

**Results:**

As the primary outcome, there was a statistically significant intergroup difference in operative test scores between the control group (mean 67.39, SD 16) and the GenAI‑assisted group (mean 77.25, SD 11.86), with the GenAI‑assisted group exhibiting higher operative test scores relative to the control group (t_110.56_=3.88, Cohen *d*=0.70; *P*<.001). For secondary outcomes, the GenAI‑assisted group also demonstrated significantly lower cognitive‑load indicators (mean 0.180, SD 0.136) than the control group (mean 0.346, SD 0.150; *P*<.001), accompanied by more efficient visual attention allocation patterns and stronger activation in prefrontal cortex, motor cortex, visual association cortex, and temporoparietal junction brain regions. The GenAI‑assisted group showed better performance across low (*P*=.003), medium (*P*=.004), and high (*P*=.004) level visual-spatial ability tests. Among low-achieving participants, the largest between-group difference in self-regulated learning was found in favor of the GenAI-assisted group (*P*=.02). Better scores were also observed for the GenAI‑assisted group in learning enjoyment, as well as behavioral, emotional, and cognitive engagement.

**Conclusions:**

Students randomized to GenAI-assisted training had higher immediate operative scores than students receiving teacher-led instruction. Secondary cognitive, behavioral, and neurophysiological findings were exploratory and pointed toward a link between GenAI exposure and variations in skill‑acquisition metrics among dental students receiving VR-based training. The results offer preliminary insights regarding the potential integration of GenAI into preclinical dental skill education.

**Trial Registration:**

Chinese Clinical Trial Registry ChiCTR2500110063; https://tinyurl.com/2hkv5wt9

## Introduction

The emergence of AI has profoundly transformed educational practices and research. AI equips systems with reasoning capabilities, enabling them to learn from experience, adapt to new inputs, and perform human-like tasks, attributes that hold significant promise for education [1]. Its applications span diverse domains with the potential to reshape instructional methods, create productive learning activities, and foster the development of technology-enhanced learning environments [2]. Indeed, AI represents an educational innovation that has been increasingly adopted to support learning across varied contexts.

Among the evolving branches of AI, generative AI (GenAI) has emerged as a particularly promising frontier. GenAI demonstrates the ability to interpret user inputs and generate personalized content, including text, code, images, audio, and video, thereby offering new modes of interaction and support [3]. In educational settings, GenAI can provide individualized, timely, and continuous feedback based on learners’ responses, effectively serving as a tutor. This not only increases students’ access to feedback but also reduces instructors’ workload, while potentially enhancing the depth and quality of the guidance provided [4].

In medical education, GenAI offers several potential benefits, such as personalizing learning experiences, simulating real-world clinical scenarios and patient interactions, and facilitating communication skills practice [5]. Notably, GenAI models like ChatGPT have demonstrated the ability to reach or approach the passing threshold on all 3 United States Medical Licensing Examination (USMLE) Step exams, even without specific fine-tuning on medical content [6]. This suggests that learners are likely to use GenAI as a quick reference and synthesis tool, enabling dynamic and interactive learning experiences beyond traditional instruction. As adult learners, medical trainees are theorized to learn most effectively when they are self-motivated, self-directed, and engaged in task-centered practice [7]. While one-on-one human tutoring is known to improve outcomes, it remains resource-intensive and often inaccessible. GenAI may offer comparable, or even superior, benefits at a fraction of the cost.

Although existing research has explored GenAI’s role in supporting theoretical knowledge and simulated interactions, competency-based medical education (CBME) emphasizes the attainment of specific competencies as key indicators of learning success [8]. Practical skills training is essential in helping students translate knowledge into clinical practice. In dental education, for instance, practical skill acquisition demands extensive practice, real-time feedback, and self-regulation. Previous studies have shown that GenAI, such as ChatGPT, has the potential to serve as an instructor for dental skills teaching [9]. While virtual reality (VR) has been increasingly integrated as a learning medium, learners in digital learning environments still require additional metacognitive support for personalized feedback, a gap where GenAI could meaningfully contribute. Although fully integrated AI-VR systems for dental education are not yet widespread, evidence suggests that AI-enhanced virtual training can improve outcomes [10]. Nevertheless, whether GenAI can effectively enhance practical medical skills training in a digital learning environment remains underexplored.

To address this gap, this study investigates the integration of GenAI into VR-based dental skill training, evaluating its impact across multiple dimensions. We focus specifically on the potential of GenAI in digital medical skills education and aim to assess its effectiveness both directly, through performance outcomes, and indirectly, through underlying cognitive and affective processes.

Visual-spatial ability is particularly critical in dental practice, directly influencing operational skill acquisition. Self-regulated learning (SRL), as a self-directed learning process, plays a vital role across disciplines. Assessing both allows for a deeper understanding of learners’ skill development. Furthermore, learning emotions and engagement serve as key mediators of the learning experience, yet they are often overlooked due to the difficulty of objective measurement. Cognitive effort and psychophysiological interactions [11] are seldom captured in detail using conventional methods. Neuroscience tools such as functional near-infrared spectroscopy (fNIRS) and eye-tracking offer a more direct and objective means of capturing learners’ real-time cognitive and attentional processes [12]. While these modalities have been applied in certain educational contexts [13,14], they have not yet been used to examine learning experiences in GenAI-supported environments. Therefore, this study uses physiological signals from the brain and the eyes to evaluate cognitive processes during GenAI-guided skill learning, thereby providing neuroscientific evidence for its efficacy.

The primary objective of this study was to analyze the effectiveness of GenAI-assisted dental skill acquisition and evaluate the associated learning experience through multiple dimensions: visual behavior, brain activation levels, visual-spatial ability, SRL capacity, learning-centered emotions, and learning engagement.

## Methods

### Participants

The study recruited 129 dental students from a top-tier Chinese university. All were in their fourth or fifth year of a 5-year dental program and had acquired theoretical knowledge, with no preclinical skills training and VR simulator operation experience. Ages ranged from 17-23 (mean 21.86, SD 0.97) years. All had normal or corrected vision.

### Experiment Design

All participants completed a 9-point eye-tracking calibration prior to the experiment, with those failing to meet the calibration standards excluded from the study. Prior to randomization, participants completed a pretest to assess their theoretical knowledge. Based on pretest scores, participants were categorized into three strata: the top 33.33%, middle 33.34%-66.66%, and bottom 66.67%-100%, representing high, medium, and low academic performance levels. Participants from each stratum were then randomly assigned to either a control group or an experimental group. Prior to the experiment, all participants completed a pretest questionnaire assessing SRL ability to establish baseline measurements.

The experiment was conducted over 7 days, involving a daily one-hour learning task. All data collection took place in a digital classroom with ambient illumination maintained between 100 and 130 lux to minimize potential interference from variable lighting conditions on experimental data. The experimental procedure consisted of 2 phases. In the first phase, spanning 6 days, participants from both groups engaged in learning using a VR simulator without any monitoring devices. The second phase occurred on the 7th day. Initially, participants wore an eye-tracker (EVERLOYAL, Wuhan, China) during continued learning sessions. They were instructed to fixate on the screen for 5 seconds before commencing a 15-minute exploratory task while using their assigned equipment. Subsequently, participants donned a wearable cap equipped with a fNIRS device (YiRuiDe). A flexible head mount was used to maintain a fixed distance between the emitters and the scalp, and optodes were adjusted until optimal signal quality was achieved. We first recorded 5 minutes of resting-state fNIRS signals. Data from the detectors were transmitted directly to a laptop, while a second laptop synchronized verbal cues, “rest,” “start,” and “complete,” with markers embedded within the recorded fNIRS data. All participants were video-recorded throughout data acquisition for retrospective artifact inspection. Following the resting-state recording, a 20-second pretask resting baseline was acquired immediately before the block paradigm. Participants then performed 6 repeated cycles of a 50-second operative task followed by a 20-second short resting interval, yielding a total operative task duration of 300 seconds. During the short resting intervals between task blocks, participants were instructed to remain still and fixate on a static marker. During the 20-second pretask baseline period, participants were instructed to remain as still as possible to minimize motion artifacts and prepare to initiate the first task block upon cue.

During the training phase, participants in both groups completed identical learning tasks on the same VR equipment. Unlimited opportunities to seek guidance were available for all participants: the control group received responses from on-site professional instructors, whereas the experimental group received feedback via ChatGPT. No AI usage was permitted outside the designated learning tasks.

After the training intervention, all participants completed posttraining assessments, including a practical operational test, visual-spatial ability test, and 3 questionnaires measuring SRL, learning emotions, and learning engagement. The operational skills test score constituted the primary outcome. Secondary exploratory outcomes comprised eye-tracking parameters, fNIRS activation signals, visual-spatial ability, SRL, learning emotions, and learning engagement.

This study adopted partial blinding. All outcome raters and data analysts were blinded to group allocation, while participants and on-site research assistants knew which intervention they received.

### Pretest Knowledge

A total of 10 open-ended questions on dental operations (memory: 4, comprehension: 3, application: 3) were formulated and scored by 3 experts (10 points/question, total 100; interrater reliability: *r*=0.87; *P*<.001).

### Operational Test

After the experiment, participants completed a 30-minute practical operation test. Two qualified and experienced experts (interrater reliability: *r*=0.89; *P*<.01) scored their performance using rubrics adapted from the Chinese Dental Licensing Examination, with total scores ranging from 0 to 100. Detailed scoring criteria are provided in Multimedia Appendix 1.

### Visual-Spatial Ability Tests

Three tests were selected to assess visual-spatial ability in this study: (1) Card Rotation Test (CRT): consisting of 8 tasks for low-level visual-spatial ability [15]. (2) Cube Comparison Test (CCT): consisting of 21 tasks for intermediate visual-spatial ability [15]. (3) Mental Rotation Test (MRT): consisting of 12 tasks for high-level visual-spatial ability [16]. Tasks in each category had to be completed within 3 minutes. Scores for each test were standardized to a range of 0-100.

### SRL Abilities Questionnaire

SRL ability was assessed using an adapted version of the Self-Regulated Learning Strategies (SRLS) scale developed by Pintrich and De Groot [17]. The questionnaire used a 5-point scale (1=“strongly disagree” to 5=“strongly agree”) and consisted of 8 items. The Cronbach α coefficient for this questionnaire was 0.812.

### Learning-Centered Emotions Questionnaire

Assessed postintervention using single 6-point Likert items (1=“very little” to 6 = “very much”) for enjoyment (positive), anger, and frustration (negative). Single items are valid, reliable [18,19], and used in digital learning research [20,21], reducing participant fatigue.

### Learning Engagement Questionnaire

To measure learning engagement, the engagement scale developed by Reeve and Tseng [22] was used, which included 17 items across three dimensions: (1) behavioral engagement, (2) cognitive engagement, and (3) emotional engagement. The Cronbach α coefficients for the 3 dimensions were 0.874, 0.796, and 0.898, respectively.

### Materials

A desktop VR system (Zhonghui, China) was used for dental skills training. This desktop virtual training system displays 3D scenes on a stereoscopic monitor; participants’ physical haptic joysticks map to digital dental instruments on screen with 1000 Hz real-time haptic feedback. The screen brightness was uniformly set to 150 nits for all participants. To analyze the distribution of visual attention during the learning process, eye-tracking analysis software was used to define four areas of interest (AOIs): the operative region (AOI 1), the instrument region (AOI 2), the literature region (AOI 3), and an irrelevant region (eg, extraneous patient information) (AOI 4). A schematic of the interface layout is presented in Figure 1.

**Figure 1:**
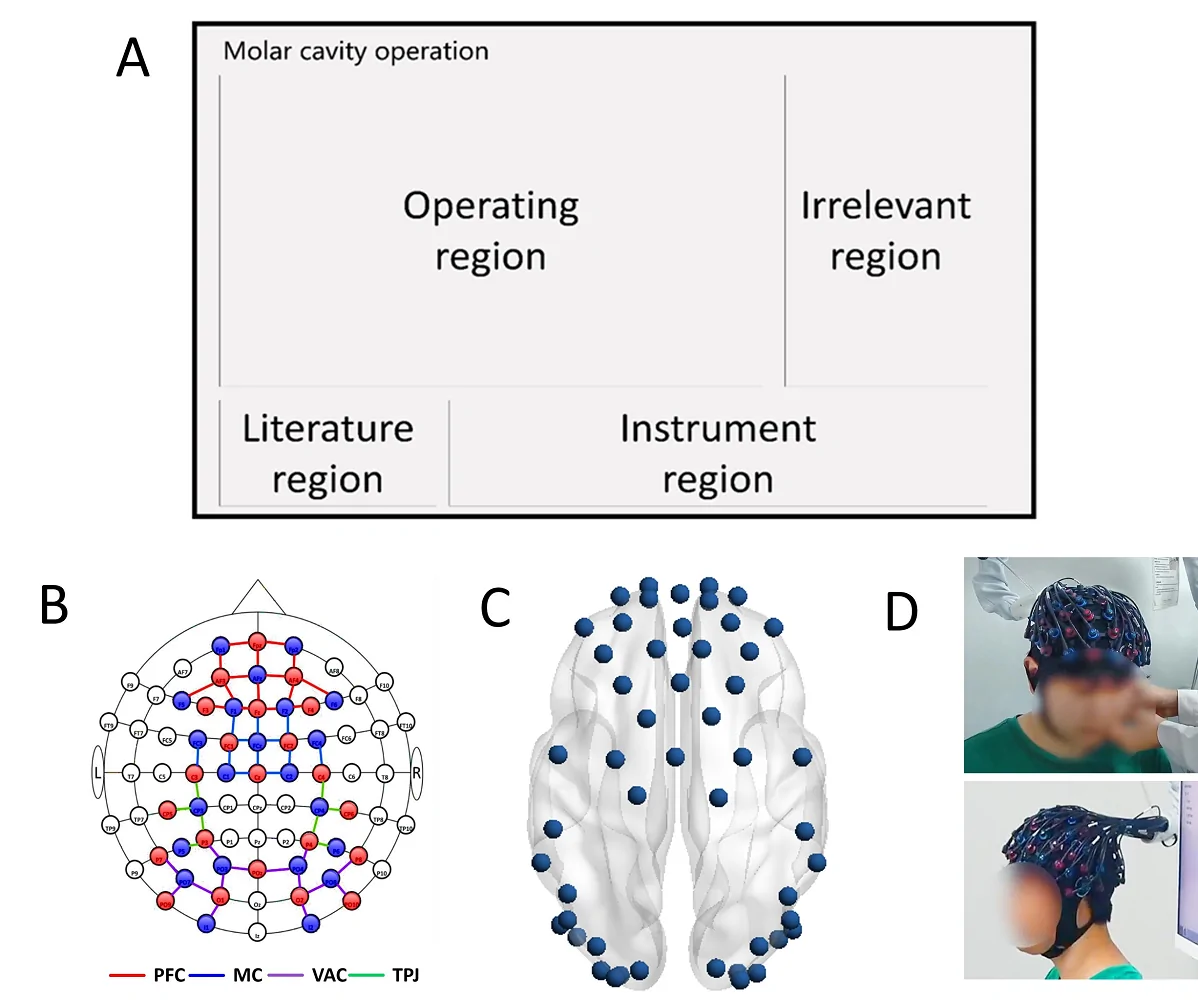
Schematic of the interface layout and distribution of functional near-infrared spectroscopy light sources, detectors, and channels. (A) Partition layout of the task display screen during virtual reality operation, divided into four functional visual subregions: operating region, irrelevant region, literature region, and instrument region. (B) Overview of the functional near-infrared spectroscopy probe cap montage. Red dots represent light sources, and blue dots represent detectors; colored lines denote measurement channels covering distinct brain regions: prefrontal cortex (PFC, red lines), motor cortex (MC, blue lines), temporoparietal junction (TPJ, green lines), and visual association cortex (VAC, purple lines). (C) The positions of the channels on a 3D cortical map. (D) Schematic of the placement of the functional near-infrared spectroscopy headset. MC: motor cortex; PFC: prefrontal cortex; TPJ: temporoparietal junction; VAC: visual association cortex.

The experimental intervention adopted ChatGPT-4.0 (OpenAI) with simple contextual training, accessed via a separate browser on independent computers isolated from the VR system. Experimental participants were provided standardized prompt templates and a unified operation guide to use ChatGPT for solving operational doubts and acquiring skill guidance. Research assistants audited AI outputs throughout training to ensure all content complied with teaching standards. The prompt templates adopted and sample ChatGPT responses are presented in Multimedia Appendix 2.

Eye movements were monitored at a sampling rate of 60 Hz using an aSee eye-tracker (EVERLOYAL), which tracked both eyes simultaneously. Before the experiment, participant positioning was calibrated to maintain a viewing distance of approximately 70 cm from the screen. Participants were instructed that they could move their heads freely during the experiment but should avoid excessive movement. The aSee eye-tracker was used for both the collection and subsequent analysis of eye movement data.

Brain activity was monitored using a fNIRS device (YiRuiDe) with a sampling frequency set at 10 Hz. The system used near-infrared light at 2 wavelengths (760 nm and 850 nm) to detect concentration changes in oxygenated hemoglobin (HbO₂). In accordance with the international 10-10 electrode placement system, the device’s 22 sources and 22 detectors were configured into 50 channels. These channels were positioned over key brain regions: the prefrontal cortex (PFC; covering Brodmann areas BA9, BA10, BA46), the motor cortex (MC; BA4, BA6, and BA8), the visual association cortex (VAC; BA18; BA19), and the temporoparietal junction (TPJ; BA39 and BA40). The fNIRS device and the spatial arrangement of its sources and detectors are illustrated in Figure 1.

### Data Analysis

For eye-tracking data, raw pupil diameter was extracted to measure cognitive load. To facilitate further signal processing, data points marked as blinks, along with 50 milliseconds of data before and after each blink, were removed, as eyelid movements during these periods may distort pupil diameter measurements. Fluctuations in pupil size were smoothed using MATLAB (MathWorks) to obtain a continuous curve of pupil size over time, as illustrated in Figure 2. In analyzing pupil diameter changes during the learning process, the median pupil size at the beginning of the learning session (serving as the baseline) and the overall median pupil size during the learning process were calculated separately. The preference for the median over the mean was driven by its greater robustness to noise and outliers. To comprehensively explore the distribution of visual attention among participants in different groups, heat maps were generated based on group assignments. Individual fixation durations were accumulated for each pixel on the screen using MATLAB programming. Differences in visual attention distribution between the 2 groups were visualized through color variations: red indicates areas of the most frequent information processing; yellow and green represent areas receiving less attention; and blue signifies the least attended regions. However, heat maps only illustrate differences in overall cumulative visual attention to specific areas during instruction and are methodologically insufficient for capturing individual differences in information searching. To investigate potential differences in cognitive processes between the GenAI and traditional teacher-led modes, this study selected three eye-tracking metrics for AOI1: (1) percentage of fixation duration within AOI1; (2) percentage of time spent in AOI1; and (3) fixation rate in AOI1. Based on the cluster analysis results, the Kruskal-Wallis H test followed by Dunn’s post hoc comparisons was used to determine whether differences existed in these 3 metrics across cluster groups. Additionally, to explore patterns of visual attention shifts, transition matrices were generated based on the different groups identified in the cluster analysis, quantifying the relative probability of shifting from one AOI to each of the remaining 3 AOIs.

**Figure 2:**
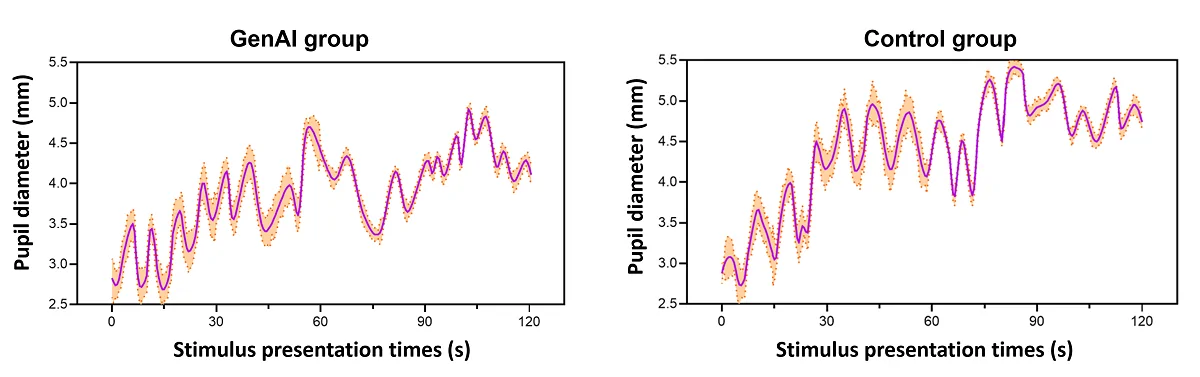
Sample smoothed pupil diameter time series curves of generative AI group and control group. GenAI: generative AI.

For fNIRS data, raw signals were preprocessed for artifact and noise reduction via six sequential steps: (1) exclusion of non-experimental time segments; (2) rejection of motion and superficial artifacts; (3) conversion of light intensity to optical density; (4) bandpass filtering (0.01-0.2 Hz) to remove drift, cardiac, and respiratory noise; (5) conversion of optical density to oxyhemoglobin (HbO) concentration; and (6) definition of the canonical hemodynamic response function (HRF) for general linear model (GLM) modeling. Task onsets were marked at the start of each of the six task blocks. A pretask baseline window was defined from −20 to 0 seconds (the 20-s resting period before the first task block). Channel-wise HbO time series per participant were modeled using a GLM. The GLM adopted the canonical HRF to estimate task-related cortical activation, producing β coefficients representing the magnitude of channel-specific activation [23]. All subsequent cortical activation analyses were based on these β values.

Normality of residuals was examined for each dependent variable. For SRL and learning-engagement variables, assumptions for parametric tests were violated; hence, Mann-Whitney *U* tests were adopted. For operational test scores, pupil diameter, visual-spatial ability, and learning-centered emotion outcomes, normality assumptions were satisfied; therefore, 2-tailed Welch-corrected independent-samples *t* tests were consistently applied for robust inference under both equal and unequal variance conditions. Spearman rank correlation coefficient (ρ) was calculated to quantify the association between visual-spatial ability and operational test scores.

To control the familywise type I error rate arising from multiple comparisons, Holm-Bonferroni correction was applied separately within each prespecified domain for secondary outcomes including visual-spatial ability tests, SRL (posttest comparisons across high-, average-, and low-achieving subgroups), learning-centered emotions, and learning engagement; this domain-wise correction strategy prevents excessive conservatism caused by correction across theoretically unrelated constructs. For cluster analyses of eye-tracking data, Kruskal-Wallis H tests followed by Dunn’s post hoc tests with Bonferroni correction were performed on eye-tracking metrics and operative test scores within each group, and these within-indicator comparisons were excluded from the cross-domain Holm-Bonferroni correction to avoid redundant double adjustment. For fNIRS data, 2-tailed cluster-based permutation *t* tests were adopted to control type I error risk across all channels, with no secondary correction implemented. Except for operative test scores and pupil diameter, all reported *P* values are adjusted *P* values.

### Ethical Considerations

The study protocol was approved by the Ethics Committee of the School and Hospital of Stomatology, Wuhan University (WDKQ2025-B71).

Written informed consent was collected from every participant prior to enrollment. All subjects joined this research voluntarily. The consent form clarified collection and subsequent analysis of eye-tracking, fNIRS, and behavioral data, as well as the potential publication of de-identified experimental screenshots within figures and tables. Participants received no compensation for participation.

All research data were deidentified immediately after collection to protect participant privacy. Study outputs are presented anonymously without any personal identifying information.

## Results

### Distribution of Participants

During the eye-tracking calibration, 6 participants were excluded because they did not pass the calibration. During the experiment, a total of 123 students participated. This study adopted a stratified randomization-based design on pretest scores; participants within each stratum were randomly assigned to either the control group (n=61) or the experimental group (n=62). The experimental group consisted of 30 males and 32 females, while the control group comprised 31 males and 30 females. The distribution of academic levels and gender was balanced across both groups to ensure the representativeness of the sample. Table S1 in Multimedia Appendix 3 presents participants’ demographic information and sample size. The detailed experimental procedure is illustrated in Figure 3.

**Figure 3:**
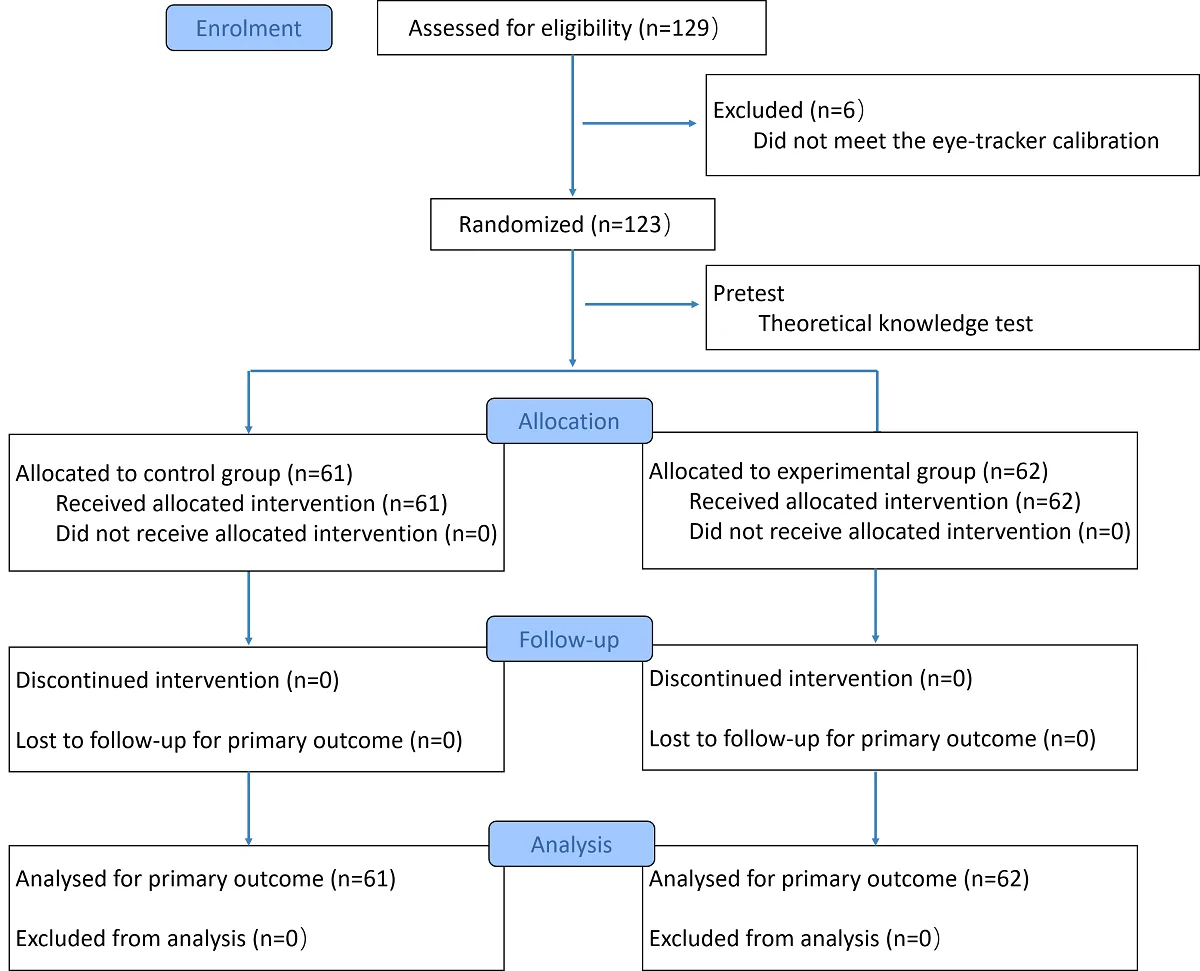
CONSORT (Consolidated Standards of Reporting Trials) flow diagram.

### Operational Test Scores

Two-tailed independent-samples Welch *t* test was used to compare operative test scores between the control group (mean 67.39, SD 16) and the GenAI-assisted group (mean 77.25, SD 11.86). The mean difference between groups was 9.86 points (95% CI 4.81-14.91). This intergroup difference was statistically significant (*t*_110.56_=3.88, Cohen *d*=0.70; *P*<.001), with the GenAI-assisted group presenting substantially higher operation test scores than the conventional control group.

### Eye-Tracking Metrics

#### Pupil Diameter

To assess and compare the cognitive load experienced by participants during the learning process, changes in pupil diameter from baseline were analyzed as a physiological proxy for cognitive load. An independent-samples Welch *t* test was used to compare the changes in pupil size between the control group (mean 0.346, SD 0.150) and the GenAI-assisted group (mean 0.180, SD 0.136). The mean difference between groups was 0.166 mm (95% CI 0.115-0.217). This intergroup difference was statistically significant (*t*_119.45_=6.43, Cohen *d* =1.16; *P*<.001), with the GenAI-assisted group exhibiting a substantially smaller increase than the control group.

#### Visual Attention Distribution

Visual attention distribution was analyzed by leveraging heat maps generated from gaze duration data, illustrating how participants in the GenAI and control groups allocated their visual attention. Figure 4 presents the heat map outputs for both groups, along with the fixation duration in different AOIs for the learning interface (translations for non-English text in subgraphs A and B are provided in Multimedia Appendix 4).

**Figure 4:**
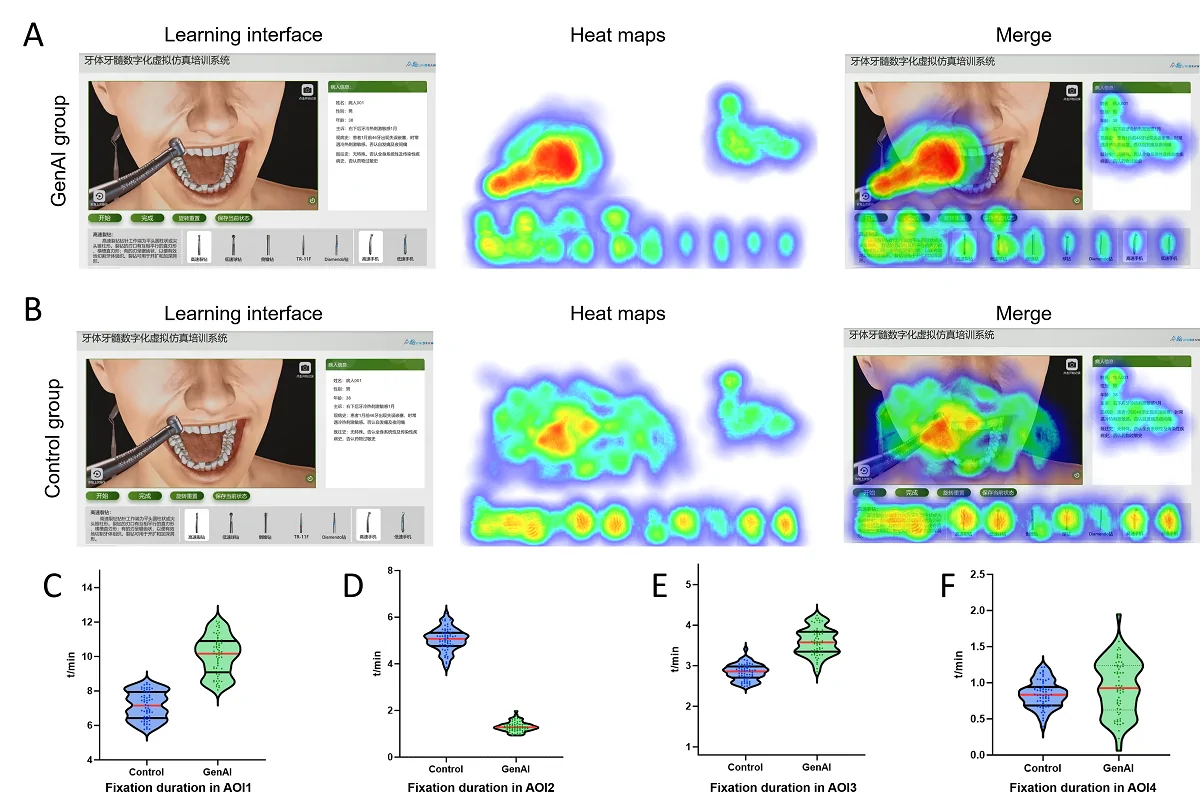
Visual attention heat maps and area of interest fixation duration of two groups. Visual attention distribution and quantitative eye-tracking indicators for the generative AI group (A) and control group (B). Each row includes the training interface, gaze heat map, and merged heat map–interface overlay; warmer heat map colors stand for longer fixation duration. Panels C-F are violin plots comparing fixation duration (unit: min) across four predefined area of interests. Both groups primarily focused on molars operative field (area of interest 1). The control group showed scattered, distracted visual attention, spending more time viewing all instruments (area of interest 2) and the documentation area (area of interest 3), whereas the GenAI group selectively attended only to procedure-relevant tools. Minimal attention was paid to irrelevant interface regions (area of interest 4) by both groups. Translations for non-English text in subgraphs A and B are provided in Multimedia Appendix 4. AOI: area of interest; GenAI: generative AI.

In the operative area, both groups exhibited significant focus on the molars and operative instruments. However, participants in the control group demonstrated a higher level of distraction. A distinct pattern emerged in the instrument area: the control group appeared to devote more attention to identifying all instruments. In contrast, participants in the GenAI group allocated only a small portion of their attention to prominent instruments, closely associated with the preparation process.

When examining the literature area, the control group showed more yellow nodes compared to the GenAI group, suggesting that the control group spent a larger proportion of time on literature-related content. Furthermore, in the irrelevant area, the presence of green and blue nodes in the GenAI and the control group indicated that participants allocated limited attention to this region.

### Cluster Analysis Based on Data in AOI 1

To further investigate individual differences in information-seeking behavior, a cluster analysis was performed on the operative area (AOI1) based on 3 key eye-tracking indices: percentage of fixation duration (PFD), percentage of time spent (PTS), and fixation rate (FR). Each of these metrics offers distinct insights into participants’ information processing within AOI1. The PFD reflects the depth of information processing in AOI1, the PTS represents the total time invested in AOI1 (including both fixations and saccades), and the FR indicates the intensity of knowledge searching within this area. As shown in Figure 5, participants from both the GenAI and control groups were clustered into 3 distinct groups. Table S2 in Multimedia Appendix 3 summarizes the values of the 3 eye-tracking indices for the GenAI and control groups across these clusters.

**Figure 5:**
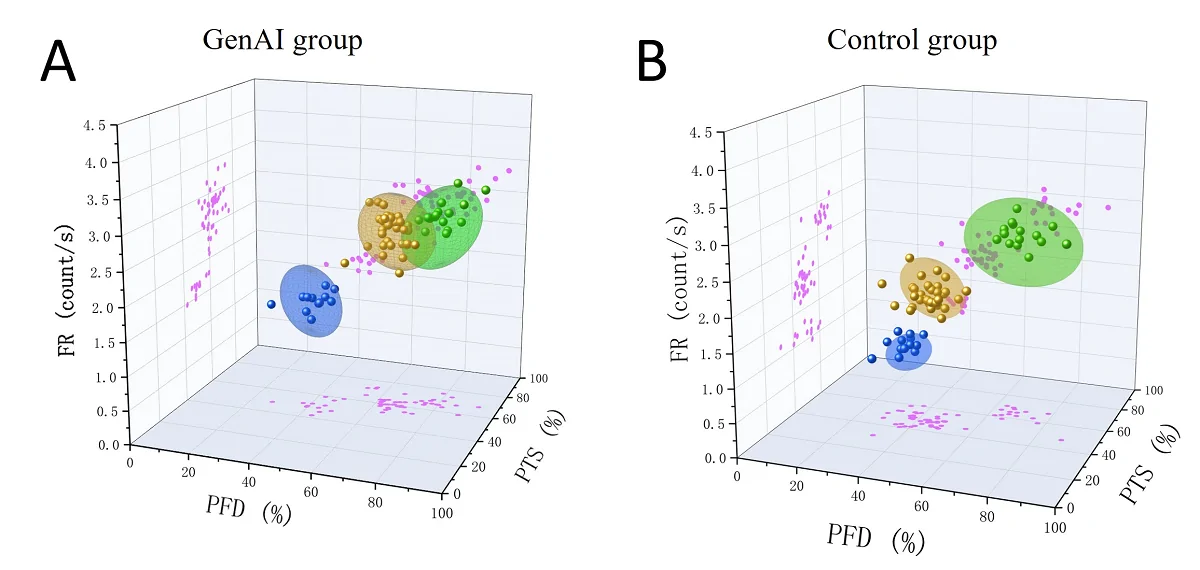
3D cluster scatter plots of three eye-tracking indicators in the operation area for two training groups. 3D cluster scatter plots visualizing three core eye-tracking indices of the operation area (area of interest 1) for the generative AI training group (panel A) and conventional control group (panel B). All participants were automatically divided into three clusters: cluster 1 (green), cluster 2 (yellow), cluster 3 (blue). Each cluster ellipsoid encloses samples belonging to the same subgroup, and scattered magenta dots represent raw individual data distribution. From cluster 3 to cluster 1, percentage of fixation duration, percentage of time spent, and fixation rate values (count/s) rise progressively, meaning cluster 1 participants achieved deeper, longer, and more frequent information search in the operation region. Overall, the generative AI group showed significantly higher percentage of fixation duration, percentage of time spent and fixation rate than the control group. FR: fixation rate; GenAI: generative AI; PFD: percentage of fixation duration; PTS: percentage of time spent.

These clustering results revealed that, regardless of whether participants learned under the GenAI‑assisted or traditional teacher-led modes, all 3 metrics gradually increased from Cluster 3 to Cluster 1, indicating that Cluster 1 processed details in AOI1 most frequently, thoroughly, and for the longest duration compared to the other clusters.

When examining differences between the GenAI-assisted and control groups, Mann-Whitney *U* tests yielded 95% CIs for the median difference of 9.22-16.74 for PFD, 4.16-10.83 for PTS, and 0.17-0.48 for FR, and confirmed that participants in the GenAI‑assisted group demonstrated significantly higher values in PFD (*U*=862, *r*=0.47; *P*<.001), PTS (*U*=1047, *r*=0.38; *P*<.001), and FR (*U*=1268, *r*=0.28; *P*<.001).

This study further examined differences in dental skill performance across the 3 cluster groups within the GenAI and control groups, respectively. Kruskal-Wallis H tests revealed that the 3 clusters differed significantly on eye-tracking metrics in both the GenAI-assisted group (*P*<.001) and the control group (*P*<.001), with large effect sizes, confirming the substantive discriminant validity of the cluster solution. As shown in Table 1, skill performance scores declined progressively from G1 to G3 in both conditions. Accordingly, Group 1 was labeled as the high-performance group (HG), Group 2 as the moderate-performance group (MG), and Group 3 as the low-performance group (LG). Dunn’s post hoc tests with Bonferroni correction revealed that, in the GenAI-assisted mode, significant differences were observed between G1 and G3 (*P*<.001; *r*=0.73), G2 and G3 (*P*=.003; *r*=0.42), and G1 and G2 (*P*=.049; *r*=0.31). In the traditional teacher-led mode, significant differences were also found between G1 and G3 (*P*<.001; *r*=0.41), G2 and G3 (*P*=.006; *r*=0.23), and G1 and G2 (*P*=.04; *r*=0.18).

**Table 1:** Results of Kruskal-Wallis tests on operation performance among the 3 groups in 2 modes.

Groups and subgroups		Mean (SD)	Mean rank	Dunn	*z*	*P* value (Dunn, Bonferroni-adjusted)	*r* (95% CI for median difference)
Experimental group^a^							
	G1	85.12 (10.18)	43.20	G1>G2	2.40	.049	0.31 (2.15-23.21)
	G2	75.25 (9.04)	31.36	G2>G3	3.27	.003	0.42 (8.44-26.02)
	G3	66.27 (9.16)	15.21	G1>G3	5.67	<.001	0.73 (15.88-42.96)
Control group^b^							
	G1	72.47 (10.31)	36	G1>G2	2.50	.04	0.18 (1.44-15.38)
	G2	65.53 (10.18)	28.65	G2>G3	3.10	.006	0.23 (2.04-17.66)
	G3	59.21 (9.89)	19.54	G1>G3	5.60	<.001	0.41 (7.15-27.24)

^a^*H*=15.85 (*P*<.001); *η*^2^*_H_*=0.235.

^b^*H*=13.50 (*P*<.001); *η*^2^*_H_*=0.198.

In summary, regardless of the instructional method used for dental skill acquisition, participants who allocated more attention to AOI1 were more likely to demonstrate superior performance. Furthermore, the GenAI group demonstrated a stronger capacity to attract and sustain student attention on AOI1.

### Transition Matrices for Visual Transitions Analysis

To visualize participants’ visual transitions between AOIs, this study used transition matrices. As illustrated in Figure 6, each matrix represents the relative probability of participants shifting their gaze from a previous AOI to a current AOI, with darker colors indicating higher transition probabilities. Significant visual transitions between AOIs were abstracted and visualized in Figure 7, an arrow pointing from A to B indicates that when a participant's gaze was currently in AOI A, they were more likely to shift their gaze to AOI B compared to the other 2 areas.

**Figure 6:**
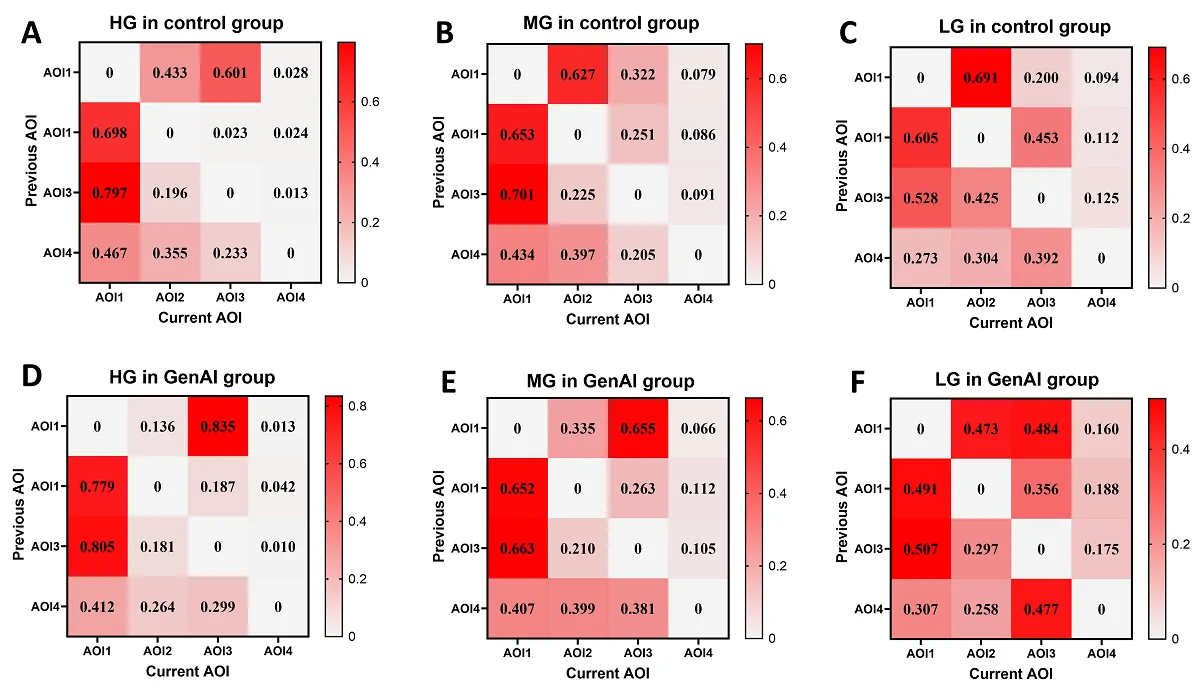
Area of interest gaze transition probability heat maps of three subgroups in two training groups. Gaze transition probability heat matrices for three performance subgroups under conventional training (panels A-C: high-performance group, moderate-performance group, and low-performance group) and generative AI–assisted training (panels D-F: high-performance group, moderate-performance group, and low-performance group). Rows represent the prior fixation area of interest, and columns represent the subsequent area of interest; deeper red shading denotes a higher gaze shift probability. All subgroups showed the highest transition probability flowing toward area of interest 1, with subgroup-specific differences in cross-region transition strengths. AOI: area of interest; GenAI: generative AI; HG: high-performance group; LG: low-performance group; MG: moderate-performance group.

By integrating Figures 6 and 7, three distinct transition patterns were identified across the 6 groups. The first pattern, observed in the HG group under the traditional teacher condition and in both the HG and MG groups under the GenAI condition, showed a tendency to shift gaze from AOI2, AOI3, and AOI4 to AOI1, along with a higher probability of gaze transitions from AOI1 to AOI3. The second pattern, represented by the MG group in the traditional teacher condition, resembled the first pattern but demonstrated an additional tendency to transition from AOI1 to AOI2. The third and final pattern was observed in the LGs under both the GenAI and traditional teacher conditions. Both variants exhibited transitions from AOI2 and AOI3 to AOI1, as well as from AOI4 to AOI3. However, when gaze transitions originated from AOI1, the LG group under the GenAI condition was more likely to shift gaze to AOI3, whereas the LG group in the traditional teacher condition tended to transition to AOI2.

**Figure 7:**
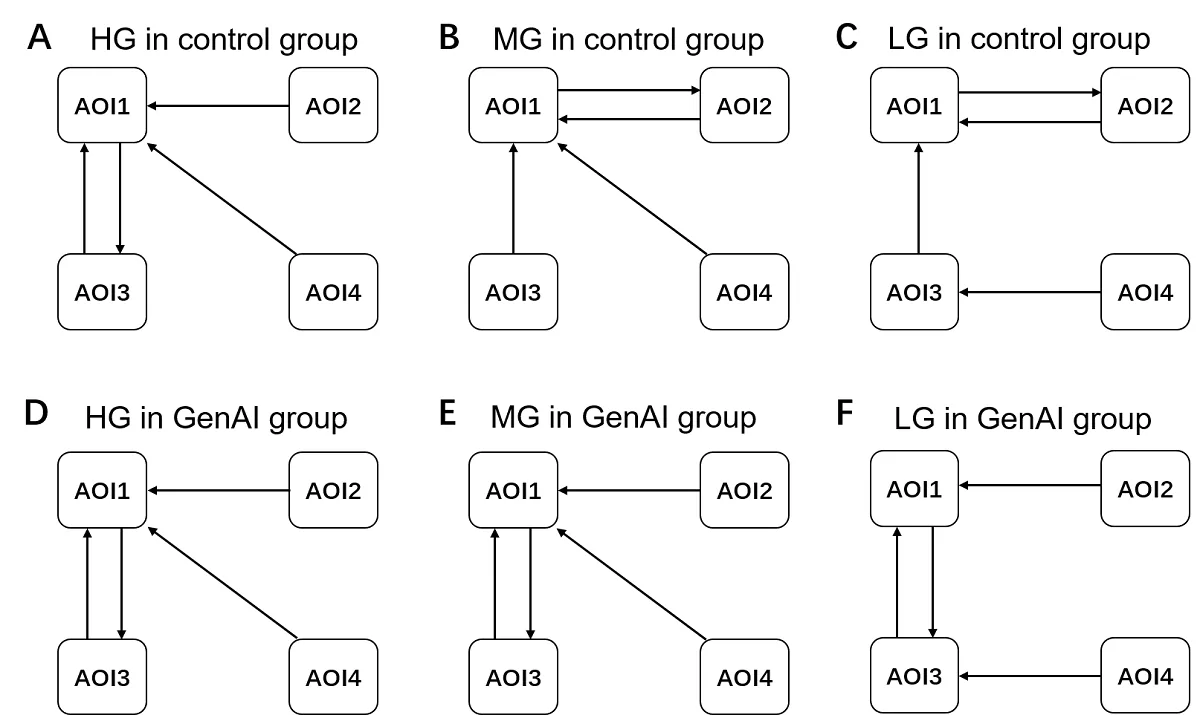
Simplified schematic diagrams of significant area of interest gaze transition paths for two training groups. Simplified diagrams of high-probability gaze transition paths for three performance subgroups in the control group (A-C) and generative AI group (D-F). Directed arrows indicate prominent gaze shift directions between four functional area of interests. Three distinct visual switching patterns were identified across all subgroups. Control high-performance group, generative AI high-performance group, and generative AI moderate-performance group primarily transferred gaze from auxiliary area of interests to the operation area of interest 1 and cycled between area of interest 1 and area of interest 3; control moderate-performance group added bidirectional switching between area of interest 1 and area of interest 2. Low-performance subgroups of both groups transferred gaze from area of interest 2 and area of interest 3 to area of interest 1 and from area of interest 4 to area of interest 3; generative AI low-performance group tended to shift gaze from area of interest 1 to area of interest 3, while control low-performance group preferred transitions from area of interest 1 to area of interest 2. AOI: area of interest; GenAI: generative AI; HG: high-performance group; LG: low-performance group; MG: moderate-performance group.

### fNIRS Analysis

The analysis focused on cortical activation in the PFC, MC, VAC, and temporoparietal junction (TPJ). Descriptive statistics of β‑values for these brain regions across the 2 groups are presented in Table S3 in Multimedia Appendix 3. Compared to baseline, the experimental group exhibited significant activation in the PFC, MC, VAC, and TPJ, with particularly pronounced activation observed in the left PFC and right TPJ. The control group also showed varying degrees of activation in the PFC, VAC, and TPJ relative to baseline. Two-tailed permutation *t* tests revealed that the experimental group had significantly higher β-values than the control group across all regions of interest, PFC, MC, VAC, and TPJ, in both hemispheres. Figure 8 provides a clear visual comparison of cortical activation patterns between the 2 groups across all examined brain regions.

**Figure 8:**
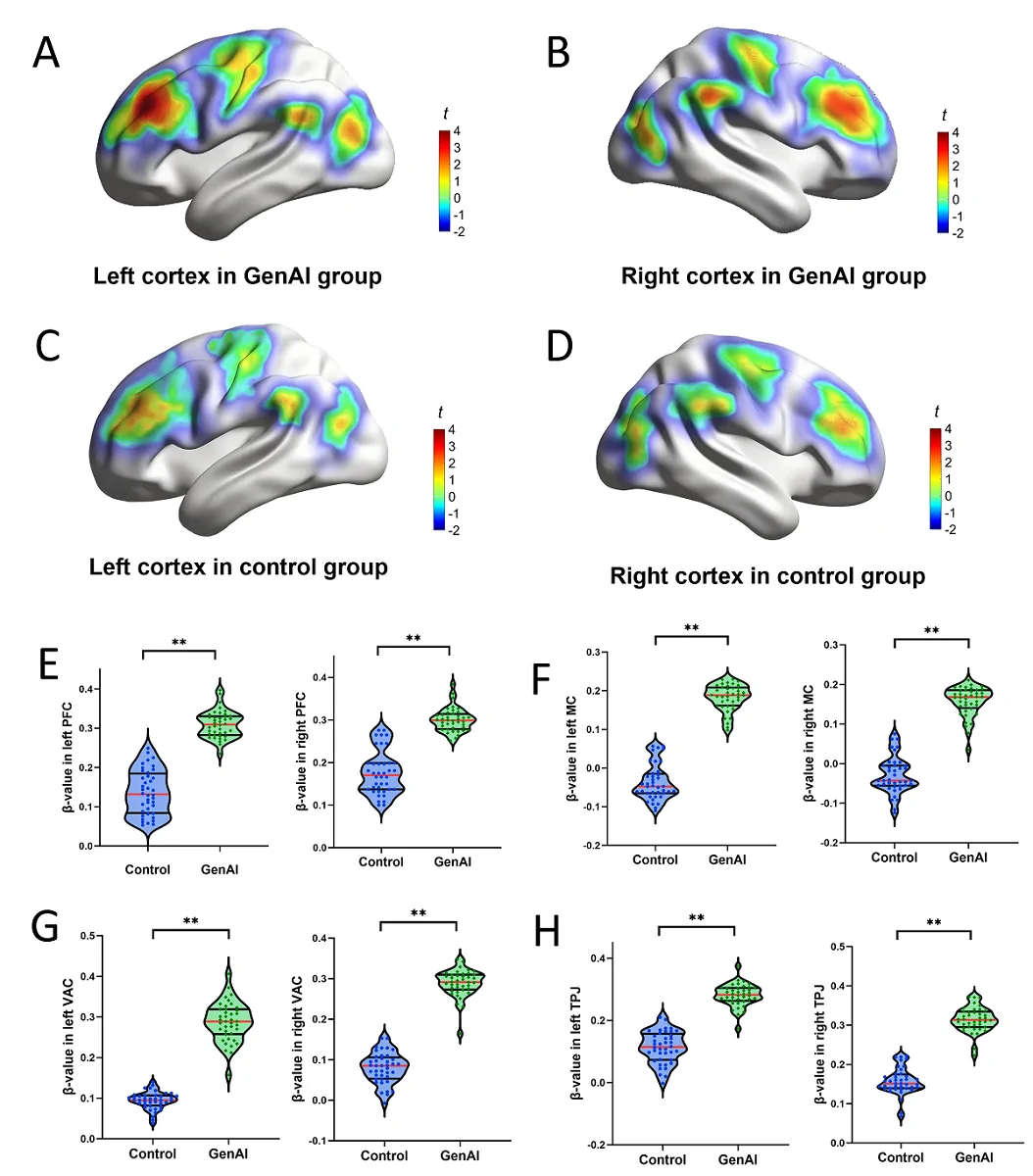
3D cortical activation maps and β-value violin plots of four brain regions for GenAI and control groups. Cortical activation t-maps (A-D) and violin plots of β coefficients (E-I) for four analyzed brain regions: prefrontal cortex, motor cortex, visual association cortex, temporoparietal junction. Panels A and B show left and right cortical activation patterns of the GenAI group; panels C and D display left and right cortical activation of the control group, with warmer colors indicating stronger cortical activation. Violin plots E-H compare bilateral β values between the two groups. The GenAI group presented significantly higher β values in all bilateral brain regions than the control group. β value is the activation regression coefficient, and t is the t statistic. MC: motor cortex; PFC: prefrontal cortex; TPJ: temporoparietal junction; VAC: visual association cortex. **P<.01.

### Visual-Spatial Ability

In both groups, accuracy followed the same descending pattern across the three levels of visual-spatial tests: highest on the low-level (CRT), followed by the intermediate-level (CCT) and the high-level (MRT). Table 2 shows that the GenAI-assisted group significantly outperformed the control group on all three tests (CRT: *P*=.003; CCT: *P*=.004; MRT: *P*=.004).

**Table 2:** Descriptive statistics and Welch-corrected independent‑samples *t* tests for visual-spatial ability tests.

Variables and groups			Minimum-maximum		Mean (SD)		*t* test (Welch *df*)			*P* value (Holm-Bonferroni adjusted)				Cohen *d* (95% CI for mean difference, Exp-Ctrl)	
Card rotation (%)								4.21 (116.79)			.003				0.76 (8.20-23.04)
	Experimental	36.43-100		77.62 (18.81)											
	Control	20.04-100		62 (22.42)											
Cube comparison (%)								3.44 (114.30)			.004				0.62 (5.52-20.60)
	Experimental	11.31-100		66.96 (23.76)											
	Control	15-95		53.90 (18.25)											
Mental rotation (%)								3.27 (116.02)			.004	0.59 (4.90-20.80)			
	Experimental	13.22-100		59.27 (24.22)											
	Control	6.53-81.03		46.42 (19.30)											

Correlation analysis within this cohort revealed differing associations between the 3 tiers of visual‑spatial ability and operative test scores. Spearman correlation analyses identified statistically significant moderate positive correlations between operative performance and both low‑level (CRT) (ρ=0.591; *P*<.05) and intermediate‑level (CCT) visual‑spatial tests (ρ=0.427; *P*<.05) in both the experimental and control groups. By contrast, no significant correlation between high‑level visual‑spatial test (MRT) performance and operative test scores was detected in either group. Correlation statistics for visuospatial‑ability and operative‑test‑score associations for the experimental and control groups are provided in Tables S4 and S5 of Multimedia Appendix 3.

### SRL Ability

Recognizing that SRL competencies may vary with academic proficiency, we performed stratified analyses comparing SRL abilities between conditions within high-, average-, and low-achieving student subgroups. Table 3 presents the results of these stratified analyses. Baseline SRL abilities did not differ significantly between the experimental and control groups in any of the 3 subgroups, supporting the comparability of the 2 conditions at baseline. In the posttest, statistically significant intergroup differences were observed for average- (*P*=.04, *r*=0.36) and low-achieving students (*P*=.02, *r*=0.42), whereas only a nonsignificant favorable trend was detected among high-achieving students (*P*=.06, *r*=0.29). Relative to the control condition, GenAI-supported learning was associated with greater SRL improvements, with effects appearing to be more pronounced among students with lower prior academic achievement.

**Table 3:** Descriptive statistics and Mann‑Whitney U test analysis for the SRL‑ability test.

Variables, academic subgroup, and groups			Baseline test									Posttest									
SRL^a^ abilities			Mean (SD)		*U*			*P* value			Mean (SD)			*U*			*P* value (Holm-Bonferroni adjusted)			*r* (95% CI for median difference, Exp-Ctrl)	
HAs^b^						226			0.881						145			.06			0.29 (–0.27 to 8.75)
	Experimental	28.98 (1.24)								32.45 (2.12)											
	Control	29.01 (1.55)								31.21 (1.95)											
AAs^c^						229			0.724						129			.04			0.36 (0.36 to 8.90)
	Experimental	23.10 (1.66)								30.36 (1.89)											
	Control	23.45 (1.73)								25.73 (2.03)											
LAs^d^						224			0.593						113			.02			0.42 (2.14 to 10.12)
	Experimental	18.51 (1.92)								26.52 (1.99)											
	Control	17.92 (2.21)								20.39 (1.92)											

^a^SRL: self-regulated learning.

^b^HA: high‑achieving student.

^c^AA: average‑achieving student.

^d^LA: low‑achieving student.

### Learning-Centered Emotions

Two-tailed independent-samples Welch *t* tests were conducted to compare three learning-centered emotion variables between the GenAI-assisted group and the control group. As shown in Table 4, the GenAI group reported significantly higher enjoyment than the control group, indicating a large effect size (Cohen *d*=0.98; *P*<.001). For negative emotions, the GenAI group exhibited significantly lower anger than the control group, also demonstrating a large effect (Cohen *d*=1.05; *P*<.001). However, only marginal statistical significance was observed in frustration between the 2 groups (*P*=.07).

**Table 4:** Descriptive statistics and Welch‑corrected independent‑samples *t* tests for learning-centered emotions.

Variables and groups		Mean (SD)	*t* test (Welch *df*)		*P* value (Holm-Bonferroni adjusted)		Cohen *d* (95% CI for mean difference, Exp-Ctrl)	
Enjoyment				5.41 (119.20)		<.001		0.98 (0.51 to 1.09)
	Experimental	4.78 (0.85)						
	Control	3.98 (0.79)						
Anger				–5.80 (120.97)		<.001		1.05 (–1.29 to 0.63)
	Experimental	2.34 (0.93)						
	Control	3.30 (0.91)						
Frustration				–1.80 (120.73)		.07		0.32 (–0.69 to 0.03)
	Experimental	1.89 (1.13)						
	Control	2.22 (0.89)						

### Learning Engagement

Regarding learning‑engagement metrics across 3 dimensions, Table 5 shows that more favorable outcomes in behavioral, emotional, and cognitive engagement were observed among students in the GenAI‑assisted group relative to the control group.

**Table 5:** Descriptive statistics and Mann‑Whitney U test analysis for learning engagement.

Variables and groups		Mean (SD)	*U*		*P* value (Holm-Bonferroni adjusted)		*r* (95% CI for median difference, Exp-Ctrl)	
Behavioral engagement				2344		.03		0.207 (0.40-5.65)
	Experimental	21.29 (1.95)						
	Control	18.54 (2.33)						
Emotional engagement				2371		.03		0.219 (0.54-5.78)
	Experimental	17.42 (1.55)						
	Control	14.78 (1.29)						
Cognitive engagement				2457		.01		0.258 (1.51-7.62)
	Experimental	28.67 (1.92)						
	Control	24.39 (2.40)						

## Discussion

### Principal Findings

In this study, participants who used ChatGPT as a learning auxiliary tool achieved higher scores on operative skill tests and exhibited lower cognitive load. Exploratory analyses further identified distinct neural and behavioral characteristics associated with ChatGPT-supported training, including focused visual attention on key operative regions and elevated activation in the PFC, MC, VAC, and TPJ. Relative to the traditional instructor-guided group, participants in the ChatGPT group reported higher self-rated outcomes in visual-spatial ability, SRL, learning enjoyment, and learning engagement. Overall, these findings indicate that ChatGPT-assisted training is associated with variations in preclinical dental skill acquisition metrics, providing potential implications for the integration of GenAI tools into dental preclinical skill training curricula.

Cognitive load theory holds that human working memory has a finite capacity; cognitive overload tends to emerge when cognitive load exceeds working‑memory limits, coinciding with lower learning efficiency. Given the observed correlation between ChatGPT exposure and higher operative skill scores in the present study, we performed exploratory analyses regarding cognitive‑load patterns among participants. Drawing on the well‑established research paradigm using task‑based pupil‑diameter fluctuation to index learners’ cognitive load in dental radiographic interpretation [24], the eye-tracking data in this study revealed smaller pupil diameter variations among participants in the GenAI group, a pattern that typically corresponds to lower cognitive load. A plausible interpretation for this observed trend is that ChatGPT may deliver timely explanatory support during VR-based skill training, which could potentially reduce the consumption of cognitive resources during learning.

According to cognitive load theory, processing redundant task‑irrelevant information increases extraneous cognitive load. Meanwhile, the seductive details effect states that visually appealing yet non‑task‑relevant elements occupy limited visual processing resources [25,26]. These theoretical perspectives imply that visual‑attention allocation may partially parallel underlying cognitive processing. Eye‑tracking heatmaps illustrate that participants under traditional instructor‑guided conditions frequently directed attention toward irrelevant instrument areas, whereas participants in the GenAI group oriented their attention toward core operative interfaces and theoretical text regions. We further examined visual‑attention indicators for AOI1 (operative zone). The results showed that sustained attention toward the operative zone correlated with better skill performance irrespective of intervention type. Analysis of visual saccadic transitions revealed a representative gaze pattern characterized by frequent attentional shifts from peripheral regions to AOI1 (operative zone), alongside repeated bidirectional saccades between AOI1 and AOI3 (theoretical text zone). This pattern was observed among medium‑ and high‑performing learners within the GenAI group and high‑performing learners in the traditional teacher‑led group; such ocular behaviors may correspond to learners engaging in integration of visual operational information and textual theoretical knowledge during learning. Remaining gaze profiles showed diffuse attention distributed across multiple visual regions. Synthesized observations point to a pattern in which ChatGPT exposure is accompanied by greater visual‑attention concentration on task‑critical areas relative to traditional instructor‑guided training, alongside lower extraneous cognitive‑load indicators, suggesting a possible association between ChatGPT use and more focused visual attention. These observations also carry potential implications for instructional designers: optimized instructional frameworks may seek to minimize extraneous load that consumes finite working‑memory capacity while fostering germane load supportive of long‑term‑memory schema construction [27,28]. Links between targeted attention allocation and task‑completion outcomes have been documented in prior work: diagnosticians demonstrate limited capacity for effective lesion screening when attention is not oriented toward diagnosis‑relevant regions [29].

fNIRS provided exploratory information about cortical activation patterns during GenAI-assisted dental skill training. The PFC is closely associated with executive functions like working memory and motor planning. The MC is responsible for the initiation, planning, coordination, and execution of voluntary movements, closely related to fine motor skills of the hand. The VAC progressively integrates and processes visual information with increasing complexity, serving as the foundation for visual recognition and spatial perception. The TPJ, particularly in the right hemisphere, is a core region for spatial attention. Significantly stronger cortical activation signals were detected in all the above brain regions in the GenAI group. While measurable intergroup disparities existed in activation patterns, these fNIRS findings remained exploratory. Greater cortical activation may reflect differences in cognitive effort, attention, task novelty, task difficulty, or other unmeasured factors. The present study cannot determine whether these activation differences represent better learning.

Numerous previous studies have identified positive correlations between visuospatial ability and surgical operative performance [30-34]. Given the observed links between ChatGPT exposure and favorable operative scores in this study, we further explored potential connections with visuospatial ability. The results showed that GenAI exposure co‑occurred with better low‑, intermediate‑, and high‑level visuospatial ability among learners within the VR environment. The magnitude of observed links between visuospatial ability and clinical performance varies by learning stage and task complexity [34], and generally exhibits stronger predictive power during the associative learning process [35]. Notably, significant correlations with operative performance were only detected for low- and intermediate-level visuospatial assessments, while high-order spatial indicators showed no meaningful correlation. This observation partially diverges from a systematic review on surgical performance predictors, which reported higher predictive validity for intermediate and high-level visuospatial tests [36]. One tentative explanatory account for this discrepancy centers on the possibility that the overall proficiency of participants in this sample had not yet reached the associative learning stage, during which high-order spatial reasoning becomes the primary correlate of operative performance.

Prior research has documented that learners with weak academic foundations generally exhibit insufficient SRL ability [37]. This limitation was also observed among participants with low pre‑test performance in both groups of this study. Among these learners, self-reported indicators of SRL were higher following GenAI exposure. Meanwhile, even learners with high pretest scores and strong self-regulatory capacity may refine their SRLS with ChatGPT support. This trend may reflect a potential instant-feedback pathway, whereby ChatGPT may provide targeted feedback on operative attempts for learners to verify their learning accuracy [38]. Learners with poor baseline self‑regulation rely more on external scaffolding and real‑time feedback to identify their learning status, a pattern that may correspond to shifts in self‑regulated‑learning capacity [39]. In addition, given the unique VR training context adopted in this study, the magnitude of this positive correlation may be associated with the possibility that ChatGPT may help reduce perceived operative cognitive disorientation in virtual environments.

Affect and cognition are closely interconnected, and emotional fluctuations may shape learners' cognitive processing and efficiency. In this study, ChatGPT exposure was associated with higher reported enjoyment and engagement. This positive association between ChatGPT use and learning enjoyment aligns with findings from existing research [40]. Behavioral engagement refers to subjective effort, task involvement, and persistence throughout learning [41]. A possible explanation for the elevated behavioral‑engagement levels observed in this study is that GenAI may provide feedback that helps learners adjust their learning strategies and plan subsequent practice steps [42]. Both cognitive and affective engagement, which refer respectively to sustained focus on operative content and emotional responses to learning modules or activities [41], were positively associated with ChatGPT exposure in our study. These findings may reflect differences in the learning experience, but the mechanisms were not directly measured.

### Limitations

This study has several limitations: only partial blinding was implemented and full double-blinding could not be achieved due to distinct training interaction modes, which may introduce subjective scoring bias; single-item scales were adopted to assess learning emotions, lacking the reliability and comprehensiveness of standardized multiitem psychological scales; merely 1-week short-term training performance was analyzed without testing long-term skill retention and measuring real clinical performance outcomes; training was restricted to basic tooth preparation, so the results cannot be generalized to complex dental operations or other training scenarios; multiple statistical comparisons across indicators were performed, and minor residual statistical inflation could not be fully eliminated even with Holm-Bonferroni correction; detailed ChatGPT usage details of participants were not recorded during the training process. Furthermore, learners may exhibit decision offloading and develop overreliance on AI feedback during practice. Although research assistants monitored AI outputs, systematic verification of every AI response at scale was not feasible within this trial.

### Future Perspectives

Future research should further evaluate the effectiveness and safety of GenAI as an auxiliary learning tool for procedural skill training. Longitudinal tracking is needed to assess long-term skill retention, clinical transfer, and independent operational performance after AI guidance is withdrawn. Training task coverage should be expanded to validate the generalizability of this AI framework across diverse procedural operations. Future work may also evaluate the safety of automated GenAI feedback and establish standardized instructor supervision mechanisms for AI-assisted practical training.

### Conclusions

This study found that in the context of digital medical skills education, learners receiving GenAI support achieved higher scores in immediate skill tests compared with learners under traditional teacher-led instruction. This between-group difference was accompanied by reduced cognitive load indicators, different visual attention patterns, and enhanced activation in relevant brain functional regions. In addition, learners in the GenAI group obtained higher self-rated scores in visuospatial ability and SRL, 2 core competencies for dental skill learning. Concurrent findings also demonstrated greater self-reported learning enjoyment and learning engagement among these learners. Collectively, within this short-term VR-based preclinical dental skills training study, learners in the GenAI-assisted group exhibited superior immediate operational performance, alongside differences in learning-process measures. These findings suggest the potential value of GenAI tools in dental skills education.

## Data Availability

The datasets used and/or analyzed during this study are available from the corresponding author upon reasonable request.

## Appendix

Multimedia Appendix 1 Scoring rubric for skill operation tests.

Multimedia Appendix 2 ChatGPT prompt templates and interaction samples.

Multimedia Appendix 3 Tables of statistical results.

Multimedia Appendix 4 Translation of non-English texts in Figure 4.

## Abbreviations

AOI: areas of interest
BA: Brodmann area
CCT: Cube Comparison Test
CRT: Card Rotation Test
fNIRS: functional near-infrared spectroscopy
GenAI: generative AI
GLM: general linear model
HG: high-performance group
HRF: hemodynamic response function
LG: low-performance group
MC: motor cortex
MG: moderate-performance group
MRT: Mental Rotation Test
PFC: prefrontal cortex
SRL: self-regulated learning
SRLS: Self-Regulated Learning Strategies
TPJ: temporoparietal junction
USMLE: United States Medical Licensing Examination
VAC: visual association cortex
VR: virtual reality
