# Supplementary material for: Effects of Generative AI–Supported Virtual Reality Dental Skill Training on Learning Performance, Visual Behavior, and Cortical Activation Among Dental Students: Stratified Randomized Controlled Trial
Source: J Med Internet Res. 2026 Oct 1;28:e90938. doi: 10.2196/90938 (PMC13673176; doi:10.2196/90938)
Supplement: Supplementary file 1 [file jmir_v28i1e90938_app1.docx]

| **Module** | **Primary Category** | **Sub-item** | **Detailed Scoring Criteria** | **Sub-score** |
| --- | --- | --- | --- | --- |
| **Cast Full Crown Preparation (Total 50 pts)** | Operational Procedures (25 pts) | Manikin Position | The mandibular dentition of the phantom head is placed horizontally, slightly higher than the operator's elbow; the operator stands at the 7/8 o'clock position on the front right of the phantom head; alternatively, the dentition forms a 45° angle with the ground, and the operator stands at the 11/12 o'clock position on the rear right | 3 |
|  |  | Grip Method & Fulcrum – | Modified pen grip for the high-speed handpiece | 1 |
|  |  |  | Fulcrum: ring finger / middle finger rested on the dental hard tissue surface | 1 |
|  |  | Instrument Selection | TF12: guide grooves for occlusal, buccal and lingual surfaces | 1 |
|  |  |  | TF12: occlusal surface preparation | 1 |
|  |  |  | TF11: buccal and lingual surface preparation | 1 |
|  |  |  | TF12: interproximal contact opening | 2 |
|  |  |  | TF12: interproximal surface preparation | 1 |
|  |  |  | TF12: finishing and polishing | 1 |
|  |  | Operational Sequence – General rules | Operational manipulation: water cooling, intermittent grinding  Preparation sequence: occlusal surface → buccolingual surfaces → interproximal surfaces → finishing | 1 |
|  |  | Operational Sequence – Occlusal preparation | Occlusal surface preparation: prepare 1 mm deep guide grooves following anatomical morphology; uniformly remove tooth structure along the guide grooves | 3 |
|  |  | Operational Sequence – Buccal preparation | Buccal surface preparation: prepare 3 guide grooves (mesial, central, distal) on the buccal axial surface, ending 0.5 mm supragingivally, with 2°–5° convergence and 0.5 mm terminal depth | 1.5 |
|  |  |  | Uniformly remove tooth structure; prepare a 0.5 mm-wide × 0.5 mm-deep supragingival chamfer margin | 1.5 |
|  |  | Operational Sequence – Lingual preparation | Lingual surface preparation: prepare 3 guide grooves (mesial, central, distal) on the lingual axial surface to eliminate undercuts; grooves end 0.5 mm supragingivally, with 2°–5° convergence and 0.5 mm terminal depth | 1.5 |
|  |  |  | Uniformly remove tooth structure; prepare a 0.5 mm × 0.5 mm supragingival chamfer | 1.5 |
|  |  | Operational Sequence – Interproximal preparation | Interproximal surface preparation: open the interproximal contact area while protecting adjacent teeth | 1 |
|  |  |  | Form a continuous 0.5 mm-wide × 0.5 mm-deep supragingival chamfer margin with the buccal and lingual margins; convergence angle 2°–5° | 1 |
|  |  | Operational Sequence – Finishing | Finishing: contour refinement; occlusal relationship examination | 1 |
|  | Preparation Outcome (25 pts) | Overall Morphology | All line angles are smooth and rounded | 2.5 |
|  |  | Occlusal Surface | Uniform 1 mm occlusal clearance | 2 |
|  |  |  | Preserves natural occlusal anatomical morphology | 2 |
|  |  |  | Functional cusp bevel formed | 1 |
|  |  | Axial Surfaces | No undercuts on any axial surface (0 point if undercut exists on any single axial surface) | 5 |
|  |  |  | Standard convergence angle: 2°–5° | 2.5 |
|  |  | Margin | 0.5 mm-wide chamfer margin, located 0.5 mm supragingivally | 2.5 |
|  |  |  | Continuous and smooth gingival margin | 2.5 |
|  |  | Adjacent Teeth | No damage to the mesial adjacent tooth | 2.5 |
|  |  |  | No damage to the distal adjacent tooth | 2.5 |

| **Module** | **Primary Category** | **Sub-item** | **Detailed Scoring Criteria** | **Sub-score** |
| --- | --- | --- | --- | --- |
| **Endodontic Access Cavity Preparation(Total 50 pts)** | Operation Process (15 **pts**) | Instrument selection | High-speed handpiece | 0.5 |
|  |  |  | Low-speed contra-angle handpiece | 0.5 |
|  |  |  | Fissure bur | 0.5 |
|  |  |  | Round bur | 0.5 |
|  |  |  | Dental explorer | 0.5 |
|  |  |  | 10° or 15° root canal files | 0.5 |
|  |  | Grip & Fulcrum | Left hand firmly holds the extracted tooth, occlusal surface upward without random rotation | 1.5 |
|  |  |  | Right hand uses modified pen grasp to hold handpiece | 1.5 |
|  |  |  | Right ring finger serves as fulcrum | 1.5 |
|  |  | Operational motion & procedure | Intermittent grinding, bur parallel to tooth long axis | 1.5 |
|  |  |  | Drill from central occlusal fossa, gradually deepen to pre-pulp cavity | 1.5 |
|  |  |  | Penetrate dentin and remove pulp chamber roof | 1.5 |
|  |  |  | Trim chamber walls & orifices to eliminate dentin shelves | 1.5 |
|  |  |  | Locate and explore root canal orifices | 1.5 |
|  | Access Cavity Outcome (35 pts) | Cavity position, outline form | Mandibular molar: Oval/rectangular outline slightly buccal to occlusal center | 2 |
|  |  |  | Maxillary molar: Triangular/oblique trapezoid outline on central fossa |  |
|  |  | residual tooth tissue mass | 5 pts: Correct position, standard outline, rounded margin, no sound tooth damage | 8 |
|  |  |  | 3 pts: Correct position, poor outline, minor damage to sound tooth tissue |  |
|  |  |  | 0 pts: Wrong position, bad outline, severe loss of sound tooth tissue |  |
|  |  | Complete removal of pulp chamber roof | 5 pts: Explorer tip cannot hooks pulp chamber roof margins | 8 |
|  |  |  | 3 pts: Explorer tip hooks partial pulp chamber roof margins |  |
|  |  |  | 0 pts: Explorer tip hook all pulp chamber roof margin |  |
|  |  | Integrity of pulp chamber morphology and pulp floor | 6 pts: Smooth chamber walls, intact pulp floor, straight canal continuity | 9 |
|  |  |  | 3 pts: Irregular walls, residual dentin shelves, worn pulp floor |  |
|  |  | Root canal orifice localization | 5 pts: All orifices exposed, No.15 file accesses each canal linearly | 8 |
|  |  |  | 3 pts: All orifices visible, straight instrument access unavailable |  |
|  |  |  | 1 pt: At least one canal orifice missed |  |
|  |  |  | 0 pts: No root canal orifices exposed |  |
