# Supplementary material for: Effects of Generative AI–Supported Virtual Reality Dental Skill Training on Learning Performance, Visual Behavior, and Cortical Activation Among Dental Students: Stratified Randomized Controlled Trial
Source: J Med Internet Res. 2026 Oct 1;28:e90938. doi: 10.2196/90938 (PMC13673176; doi:10.2196/90938)
Supplement: Supplementary file 2 [file jmir_v28i1e90938_app2.docx]

- **Template for Maxillary First Molar Access Cavity Preparation：**
- **General Template**

You are a professional preclinical dental skills instructor. I am practicing pulp access opening of maxillary first molar in preclinical training. My operation question: [Fill in your specific question]. Please answer in accordance with standardized preclinical root canal treatment guidelines, provide step-by-step procedures for access cavity preparation/ pulp chamber refinement and root canal exploration/ explain corresponding pulpal anatomical principles/ instrument selection criteria/ bur entry angles/ standardized operation requirements.

- **Specific Template List**

（1）Pulp Cavity Anatomy

1. What are the standard pulp cavity morphology, pulp chamber height and 2. anatomical features of a maxillary first molar?

Describe the root canal system of maxillary first molar, including the quantity and pathway of mesiobuccal, distobuccal and palatal root canals.

3. What is the anatomical location, incidence and identifying evidence of the MB2 canal?

4. What are the anatomical landmarks on the pulp floor and the connecting rule of all root canal orifices of maxillary first molar?

（2）Instrument and Bur Selection

1. Which high-speed and low-speed burs are required for the whole access opening procedure of maxillary first molar, and what are their respective functions?

2. Which burs are used for initial occlusal penetration, complete pulp chamber roof removal and pulp wall refinement separately?

3. What specialized tools are applied to explore canal orifices and clear calcified dentin?

4. What are the differences in usage scenarios between endodontic files and diagnostic probes during access preparation?

（3）Entry Position, Angles and Fulcrum Control

1. How to locate the standard initial bur entry point for maxillary first molar access cavity, and what is its anatomical basis?

2. What is the bur penetration direction and the required angle relative to the tooth long axis at the initial drilling stage?

3. How to establish a stable finger fulcrum and maintain steady hand control when operating on maxillary posterior teeth?

4. How to adjust the handpiece angle to match the natural curvature of each root canal during orifice exploration?

（4）Standard Operating Procedure

1. List the full operational sequence of maxillary first molar access cavity preparation from occlusal marking, penetration, roof removal to pulp chamber finishing.

2. What criteria confirm complete removal of the pulp chamber roof, and how to avoid residual dentin overhangs?

3. What are the key trimming steps to create unobstructed straight-line access to every root canal orifice?

4. What is the standardized order to systematically inspect all root canal orifices after finishing the access cavity?

（5）Pulp Chamber Trimming & Straight-Line Access Standards

1. Define straight-line access in endodontics, and explain how to achieve unblocked access on maxillary first molar.

2. What is the proper trimming range and technique to remove cervical dentin projections inside the pulp chamber?

3. How to shape the mesiobuccal pulp wall to prevent instrument binding caused by curved root canals?

4. What morphological outline, extension range and occlusal boundary limits should a standard endodontic access cavity have?

（6）Anatomical & Clinical Principles

1. Why should the access cavity be slightly offset toward the palatal side on maxillary first molar from an anatomical perspective?

2. What biological and endodontic rationales support full removal of the entire pulp chamber roof?

3. What core benefits does straight-line access bring to subsequent root canal cleaning and shaping?

4. What anatomical factors cause the MB2 canal to sit mesially and buccally?

（7）Inspection Criteria

1. Which key aspects should be checked to verify qualified access cavity and pulp chamber morphology after preparation?

2. How to confirm the pulp chamber roof is fully eliminated with no residual dentin bridges?

3. How to check that all four root canal orifices are fully exposed without any access obstacles?

4. What is the standardized sequence for comprehensive self-inspection once access preparation is finished.

- **Complete Usage Example**

You are a professional preclinical dental skills instructor. I am practicing pulp access opening of maxillary first molar in preclinical training. My operation question: How to locate the standard entry point for maxillary first molar access opening? Please answer in accordance with standardized preclinical root canal treatment guidelines, provide standardized operation requirements and explain corresponding pulpal anatomical principles.

- **Actual sample ChatGPT response excerpts (representative examples)**

| **Student questions** | **Excerpt of Corresponding ChatGPT Response** | **Instructor Verification (Safety & Teaching Compliance)** |
| --- | --- | --- |
| What full set of handpieces, burs and hand tools do I need to prepare before starting maxillary first molar access work, and what is the purpose of each tool? | Required tools: high-speed handpiece, low-speed contra-angle handpiece, round bur, fissure bur, dental explorer, #10 and #15 root canal files. Round bur for initial occlusal penetration; fissure bur for trimming pulp chamber walls; explorer to search hidden canal openings; small files to test smooth instrument access into root canals. | Met standardized dental skill teaching protocols; no unsafe operation cues. |
| How should I hold the handpiece and set a stable finger rest when drilling maxillary molars on extracted teeth? | Use modified pen grasp for the handpiece. Fix the extracted tooth firmly with the left hand and keep its occlusal surface upward without twisting. Build a fulcrum with the right ring finger on hard tooth tissue to stabilize drilling force. | Met standardized dental skill teaching protocols; no unsafe operation cues. |
| What grinding rhythm should I follow during drilling? Is there a fixed angle between the bur and the tooth’s long axis? | Use intermittent grinding with continuous water irrigation to avoid overheating. The bur must stay parallel to the tooth long axis all the time to prevent perforation of pulp chamber walls. | Met standardized dental skill teaching protocols; no unsafe operation cues. |
| Where do I start drilling on the occlusal surface, and what gradual steps should I take to reach the pulp cavity safely? | Start drilling at the central fossa of the occlusal surface. Drill slowly to pass through enamel and dentin to form a shallow pre-pulp space before fully entering the pulp chamber. | Met standardized dental skill teaching protocols; no unsafe operation cues. |
| After entering the pulp cavity, what two trimming tasks do I need to complete for the inner chamber space? | First trim uneven pulp chamber walls; second clean dentin shelves around root canal openings, so there are no protrusions blocking instrument movement. | Met standardized dental skill teaching protocols; no unsafe operation cues. |
| What overall shape should the finished access hole on a maxillary first molar look like? How should the edge of the cavity be handled? | The access cavity takes an oblique trapezoid or triangular shape centered on the occlusal central fossa; all cavity margins need to be smoothly rounded without sharp corners. | Met standardized dental skill teaching protocols; no unsafe operation cues. |
| How can I check whether I have completely removed the entire pulp chamber roof without leftover dentin? | Slide the tip of a dental explorer along the inner edge of the cavity. If the hook of the explorer cannot catch any residual hard tissue, the pulp roof is fully removed. | Met standardized dental skill teaching protocols; no unsafe operation cues. |
| What signs show that my pulp chamber inner walls and pulp floor are well-trimmed without damage? | Qualified state: smooth inner chamber walls, intact pulp floor without wear, and unobstructed straight channels leading to each root canal. If walls are jagged or the pulp floor is ground thin, the trimming is unqualified. | Met standardized dental skill teaching protocols; no unsafe operation cues. |
| How do I judge if all root canal openings of maxillary first molar are fully exposed for smooth instrument entry? | Standard effect: all four canal orifices can be clearly seen, and a #15 root canal file can slide straight into each canal without being blocked. If one or more openings are hidden, further trimming is needed. | Met standardized dental skill teaching protocols; no unsafe operation cues. |
| What will happen if I grind away too much intact tooth tissue when opening the pulp cavity, and how can I avoid over-cutting healthy dentin? | Excessive removal of sound tooth tissue damages tooth structure and distorts the standard access outline. Control drilling depth by relying on tactile feedback and stop immediately once entering the pulp space; only trim excess dentin around the chamber without grinding deep into the pulp floor. | Met standardized dental skill teaching protocols; no unsafe operation cues. |

- **Template for Mandibular First Molar Metal Full Crown Preparation**
- **General Template**

You are a professional preclinical dental skills instructor. I am practicing full crown preparation for mandibular first molar in preclinical training. My operation question: [Fill in your specific question]. Please answer in accordance with unified preclinical dental operation standards, provide step-by-step complete operating procedures/ explain the corresponding clinical principles/ instrument selection criteria/ standardized operation requirements.

- **Specific Template List**

（1）Selection of Instruments and Burs

1. What full set of burs are required for full crown preparation of mandibular first molar, and the function of each bur?

2. Which shoulder burs match different shoulder widths respectively?

3. What instruments are used for occlusal reduction, proximal preparation and fine polishing respectively?

4. Difference in applicable scenarios between abrasive discs and fine-grit burs for proximal trimming.

（2）Selection of Instruments and Burs

1. Standard finger fulcrum types for mandibular posterior tooth preparation and matched fulcrums for different operating areas?

2. Inclination angle of the handpiece relative to the tooth long axis during buccal and lingual shoulder preparation?

3. Standard cutting technique for trimming tooth morphology.

（3）Dentin/Tooth Reduction Thickness, Shoulder Dimensions, Occlusal Clearance

1. Standard grinding thickness for buccal, lingual, occlusal, mesial and distal surfaces?

2. Standard width, subgingival depth and design principle of subgingival shoulder?

3. Standard reserved occlusal clearance and on-site self-inspection methods.

（4）Step-by-Step Procedure

1. Complete operating sequence of full crown preparation for mandibular first molar?

2. Whether shoulder preparation is carried out before or after occlusal reduction, and the clinical basis?

3. Full procedures of marginal trimming and fine polishing after tooth preparation.

（5）Clinical Restoration Principles

1. Clinical significance of fabricating a complete continuous shoulder for metal full crowns?

2. Respective applicable scenarios and advantages of subgingival and supragingival shoulders?

3. Necessity of maintaining a stable fulcrum and cutting tooth tissue evenly.

（6）Inspection criteria

1. Visual methods to judge whether the shoulder width meets the standard?

2. How to check that proximal surfaces have no protrusions or axial undercuts?

3. Overall self-inspection sequence after full crown preparation.

（7）Tooth Contouring and Margin Trimming

1. How to trim buccal and lingual surfaces to retain natural physiological convexity?

2. Standard treatment for marginal flash and burrs after preparation?

3. Retention requirements for basic morphology of occlusal cusps and fossae.

- **Complete Usage Example**

You are a professional preclinical dental skills instructor. I am practicing full crown preparation for mandibular first molar in preclinical training. My operation question: What is the standard grinding thickness for buccal and lingual surfaces? Please answer in accordance with unified preclinical dental operation standards, provide standardized operation requirements and explain the corresponding clinical principles.

- **Actual sample ChatGPT response** **excerpts (representative examples)**

| **Student questions** | **Excerpt of Corresponding ChatGPT Response** | **Instructor Verification (Safety & Teaching Compliance)** |
| --- | --- | --- |
| How should I place the phantom model and stand beside it to operate mandibular molar preparation comfortably and standardly? | Two standard placement ways: 1) Lay the mandibular dentition horizontally a little higher than your elbow, stand at the phantom’s right front 7/8 o’clock position; 2) Tilt the dentition to form a 45° angle with the ground, stand at the phantom’s right rear 11/12 o’clock position. | Met standardized dental skill teaching protocols; no unsafe operation cues. |
| What handpiece holding style is required for tooth preparation? Where should I set my finger rest during all grinding steps? | Hold the high-speed handpiece with modified pen grip. Build a stable fulcrum using your ring finger or middle finger on hard tooth tissue inside the mouth every time you grind. | Met standardized dental skill teaching protocols; no unsafe operation cues. |
| There are TF11 and TF12 diamond burs; please tell me all preparation steps each bur is used for separately. | TF12 bur: mark depth guide grooves on occlusal, buccal and lingual surfaces, occlusal reduction, open interproximal contact points, trim interproximal walls, final contour finishing and polishing. TF11 bur is only used for trimming buccal and lingual axial tooth surfaces. | Met standardized dental skill teaching protocols; no unsafe operation cues. |
| What general rules do I need to follow during grinding? What fixed order should I finish all tooth surfaces? | Always use continuous water cooling and grind intermittently to prevent pulp thermal injury. Standard preparation sequence: occlusal surface → buccal & lingual axial surfaces → interproximal areas → marginal finishing. | Met standardized dental skill teaching protocols; no unsafe operation cues. |
| How deep should the guide grooves on the occlusal surface be cut? How much clearance should I reserve for occlusion? Do I need to keep the original tooth occlusal shape? | Cut 1 mm deep occlusal guide grooves and reserve uniform 1 mm occlusal clearance. Retain the tooth’s natural cusp and fossa anatomy, and form a functional cusp bevel after reduction. | Met standardized dental skill teaching protocols; no unsafe operation cues. |
| How many guide grooves should I make on the buccal surface? Where do the grooves terminate? What taper and marginal form do I need to create on the buccal wall? | Three guide grooves (mesial, central, distal) on the buccal wall, ending 0.5 mm above the gum line. Keep axial wall taper between 2°–5°, and make a 0.5 mm wide × 0.5 mm deep chamfer margin above gingiva. | Met standardized dental skill teaching protocols; no unsafe operation cues. |
| What are the requirements for guide grooves, wall taper and marginal shape when trimming the lingual surface? | Three mesial, central and distal guide grooves on lingual wall, stopping 0.5 mm supragingivally to remove undercuts. Maintain 2°–5° axial convergence and fabricate a 0.5 mm × 0.5 mm supragingival chamfer margin. | Met standardized dental skill teaching protocols; no unsafe operation cues. |
| What two key points should I pay attention to when trimming the interproximal contact area between teeth? | First, gently open the contact gap without scratching the enamel of adjacent teeth; second, connect the buccal and lingual chamfer margins into a smooth continuous ring and keep 2°–5° wall convergence. | Met standardized dental skill teaching protocols; no unsafe operation cues. |
| After finishing all grinding work, what details do I need to check on the prepared tooth surface one by one? | Check all axial line angles are smoothly rounded; no undercuts exist on any axial wall; the chamfer margin is complete, smooth and sits 0.5 mm above gingiva; no scratch damage on mesial and distal neighboring teeth. | Met standardized dental skill teaching protocols; no unsafe operation cues. |
| What negative impact will axial undercuts bring to the final crown, and how do I avoid undercuts during preparation? | Undercuts block the crown from seating fully. Control axial wall taper within 2°–5° during grinding and check the tooth from multiple angles to eliminate concave undercut structures on every axial surface. | Met standardized dental skill teaching protocols; no unsafe operation cues. |
