# Supplementary material for: Effects of Generative AI–Supported Virtual Reality Dental Skill Training on Learning Performance, Visual Behavior, and Cortical Activation Among Dental Students: Stratified Randomized Controlled Trial
Source: J Med Internet Res. 2026 Oct 1;28:e90938. doi: 10.2196/90938 (PMC13673176; doi:10.2196/90938)
Supplement: Supplementary file 3 [file jmir_v28i1e90938_app3.docx]

Table S1 Demographic information of the participants, number of participants (% of total)

|  | Experimental group | Control group | Total |
| --- | --- | --- | --- |
| Gender | | | |
| Male | 30 | 31 | 61 |
| Female | 32 | 30 | 62 |
| Total | 62 | 61 | 123 |
| Pretest performance | | | |
| High  achievement levels | 21 | 20 | 41 |
| Average achievement levels | 20 | 21 | 41 |
| Low  achievement levels | 21 | 20 | 41 |
| Total | 62 | 61 | 123 |

Table S2 Results of Kruskal-Wallis tests on three eye-tracking indices among the clustering groups in the two modes.

| Mode | Indices | Group | Mean | *SD* | *H* | Dunn |
| --- | --- | --- | --- | --- | --- | --- |
| Experimental group | PFD | G1 | 79.83 | 8.44 | 54.98** | G1＞G2** |
|  |  | G2 | 63.76 | 2.81 |  | G1＞G3** |
|  |  | G3 | 41.66 | 1.59 |  | G2＞G3** |
|  | PTS | G1 | 66.85 | 5.64 | 46.92** | G1＞G2* |
|  |  | G2 | 61.40 | 2.00 |  | G1＞G3** |
|  |  | G3 | 50.19 | 1.90 |  | G2＞G3** |
|  | FR | G1 | 2.92 | 0.13 | 48.27** | G1＞G3** |
|  |  | G2 | 2.66 | 0.05 |  | G2＞G3** |
|  |  | G3 | 1.65 | 0.13 |  |  |
| Control group | PFD | G1 | 69.37 | 7.31 | 55.67** | G1＞G2** |
|  |  | G2 | 43.86 | 6.11 |  | G1＞G3** |
|  |  | G3 | 34.95 | 5.32 |  | G2＞G3** |
|  | PTS | G1 | 63.51 | 6.92 | 41.68 ** | G1＞G2** |
|  |  | G2 | 48.14 | 5.54 |  | G1＞G3** |
|  |  | G3 | 45.13 | 6.68 |  | G2＞G3** |
|  | FR | G1 | 2.75 | 0.65 | 57.84 ** | G1＞G2** |
|  |  | G2 | 1.93 | 0.61 |  | G1＞G3** |
|  |  | G3 | 1.13 | 0.06 |  | G2＞G3** |
| **p* < .05, ***p* < .001 | | | | | | |

Table S3 Descriptive statistical data of β-values in different brain regions between the two groups

| Brain partitions | | Experimental group | | Control group | |
| --- | --- | --- | --- | --- | --- |
|  |  | Average value | Standard deviation | Average value | Standard deviation |
| PFC | Left | 0.302 | 0.045 | 0.172 | 0.047 |
|  | Right | 0.284 | 0.040 | 0.168 | 0.039 |
| MC | Left | 0.178 | 0.038 | －0.037 | 0.046 |
|  | Right | 0.164 | 0.044 | －0.040 | 0.042 |
| VAC | Left | 0.298 | 0.053 | 0.102 | 0.032 |
|  | Right | 0.288 | 0.065 | 0.090 | 0.041 |
| TPJ | Left | 0.273 | 0.047 | 0.124 | 0.052 |
|  | Right | 0.301 | 0.042 | 0.154 | 0.049 |

Table S4 Relation between visual-spatial ability tests and operational test score in the Experimental group

| Experimental | Operational test | |
| --- | --- | --- |
| Rotation card | Rho | 0.519 |
|  | *p* | 0.042* |
| Cube comparison | Rho | 0.427 |
|  | *p* | 0.034* |
| Mental rotation | Rho | 0.138 |
|  | *P* | 0.235 |
| **p* < .05, ***p* < .001 | | |

Table S5 Relation between visual-spatial ability tests and operational test score in the Control group

| Control | Operational test | |
| --- | --- | --- |
| Rotation card | Rho | 0.423 |
|  | *p* | 0.024* |
| Cube comparison | Rho | 0.448 |
|  | *p* | 0.044* |
| Mental rotation | Rho | 0.056 |
|  | *p* | 0.369 |
| **p* < .05, ***p* < .001 | | |
