# Supplementary material for: Effects of Generative AI–Supported Virtual Reality Dental Skill Training on Learning Performance, Visual Behavior, and Cortical Activation Among Dental Students: Stratified Randomized Controlled Trial
Source: J Med Internet Res. 2026 Oct 1;28:e90938. doi: 10.2196/90938 (PMC13673176; doi:10.2196/90938)
Supplement: Supplementary file 4 [file jmir_v28i1e90938_app4.docx]

\## Top Title

Digital Virtual Simulation Training System for Operative Dentistry & Endodontics

\## Right Side Panel

Patient Information:

Name: Patient 001

Gender: Male

Age: 38

Chief Complaint: Thermal sensitivity of right mandibular posterior teeth for 1 month

Present Dental History: Patient experienced food impaction on tooth 46 one month ago, accompanied by frequent cold and hot stimulus sensitivity. No spontaneous pain or nocturnal pain reported.

Medical History: Unremarkable. No systemic or infectious diseases, no known drug allergies.

\## Labels on 3D View Buttons

Lower left corner ：Click to start recording

Upper right corner ：Restore previous operation

\## Bottom Functional Buttons

Start / Complete / Rotate & Reset / Save Current State

\## Bottom Description

High-speed Fissure Bur:

The working end of a high-speed fissure bur is flat cylindrical or pointed conical. Its cutting blades feature parallel straight cutting edges or horizontal grooved straight edges; some blades are serrated to cut dental hard tissue efficiently. Fissure burs are used for expanding and deepening cavity preparations.

\## Instrument Icon Labels

High-speed fissure bur

Low-speed round bur

Inverted cone bur

TR-11F bur

Diamendo bur

High-speed handpiece

Low-speed contra-angle handpiece
